# Spatial overlap links seemingly unconnected genotype-matched TB cases in rural Uganda

**DOI:** 10.1371/journal.pone.0192666

**Published:** 2018-02-13

**Authors:** Gabriel Chamie, Midori Kato-Maeda, Devy M. Emperador, Bonnie Wandera, Olive Mugagga, John Crandall, Michael Janes, Carina Marquez, Moses R. Kamya, Edwin D. Charlebois, Diane V. Havlir

**Affiliations:** 1 Division of HIV, Infectious Diseases and Global Medicine, University of California, San Francisco, California, United States of America; 2 Curry International Tuberculosis Center, Division of Pulmonary and Critical Care Medicine, University of California, San Francisco, United States of America; 3 Makerere University-University of California, San Francisco (MU-UCSF) Research Collaboration, Kampala, Uganda; 4 California Department of Public Health, Richmond, California, United States of America; 5 Department of Medicine, School of Medicine, Makerere University College of Health Sciences, Kampala, Uganda; 6 Center for AIDS Prevention Studies, Department of Medicine, University of California, San Francisco, United States of America; University of Otago, NEW ZEALAND

## Abstract

**Introduction:**

Incomplete understanding of TB transmission dynamics in high HIV prevalence settings remains an obstacle for prevention. Understanding where transmission occurs could provide a platform for case finding and interrupting transmission.

**Methods:**

From 2012–2015, we sought to recruit all adults starting TB treatment in a Ugandan community. Participants underwent household (HH) contact investigation, and provided names of social contacts, sites of work, healthcare and socializing, and two sputum samples. *Mycobacterium tuberculosis* culture-positive specimens underwent 24-loci MIRU-VNTR and spoligotyping. We sought to identify epidemiologic links between genotype-matched cases by analyzing social networks and mapping locations where cases reported spending ≥12 hours over the one-month pre-treatment. Sites of spatial overlap (≤100m) between genotype-matched cases were considered potential transmission sites. We analyzed social networks stratified by genotype clustering status, with cases linked by shared locations, and compared network density by location type between clustered vs. non-clustered cases.

**Results:**

Of 173 adults with TB, 131 (76%) were enrolled, 108 provided sputum, and 84/131 (78%) were MTB culture-positive: 52% (66/131) tested HIV-positive. Of 118 adult HH contacts, 105 (89%) were screened and 3 (2.5%) diagnosed with active TB. Overall, 33 TB cases (39%) belonged to 15 distinct MTB genotype-matched clusters. Within each cluster, no cases shared a HH or reported shared non-HH contacts. In 6/15 (40%) clusters, potential epidemiologic links were identified by spatial overlap at specific locations: 5/6 involved health care settings. Genotype-clustered TB social networks had significantly greater network density based on shared clinics (p<0.001) and decreased density based on shared marketplaces (p<0.001), compared to non-clustered networks.

**Conclusions:**

In this molecular epidemiologic study, links between MTB genotype-matched cases were only identifiable via shared locations, healthcare locations in particular, rather than named contacts. This suggests most transmission is occurring between casual contacts, and emphasizes the need for improved infection control in healthcare settings in rural Africa.

## Introduction

Tuberculosis (TB) remains a leading cause of death worldwide, despite the availability of effective antibiotics for drug-sensitive TB for decades, in part due to ongoing transmission from undiagnosed disease.[1] Incomplete understanding of TB transmission dynamics in high HIV prevalence settings, such as sub-Saharan Africa, remains an obstacle to optimizing TB prevention efforts. An improved understanding of where TB transmission is occurring can serve as a platform for developing approaches to interrupt transmission and reduce the burden of undiagnosed TB using active TB case finding strategies, such as community-based case finding at local transmission hotspots and contact investigation.

Molecular epidemiologic studies from sub-Saharan Africa have consistently found high rates of *Mycobacterium tuberculosis* (MTB) genotypic clustering, suggesting that most incident TB disease is a result of rapid progression to disease following recent transmission.[2–4] Despite this observation, epidemiologic links between genotype-clustered cases have been challenging to identify, thereby obscuring our understanding of how TB is spreading in sub-Saharan Africa and limiting the development of approaches that reach high-risk contacts of TB cases.[5–7] One targeted approach recommended by the WHO, household contact investigation, can identify high-risk contacts.[8] However, molecular epidemiologic data suggest that most TB transmission events in African communities occur outside of the home.[9, 10] Determining where transmission is occurring outside of the home has proven elusive in high TB and HIV prevalence settings.

We sought to address this knowledge gap by conducting a molecular epidemiologic study of all adults starting TB treatment in a rural Ugandan township over three years, collecting information from each TB patient on social contacts and the geographic locations frequented prior to starting treatment, and characterizing potential epidemiologic links between genotype-clustered TB patients.

## Methods

From July 2012 through July 2015, we sought to recruit and enroll all adult (≥18 years) residents initiating TB treatment in Tororo municipality, Uganda. Tororo is a rural township in eastern Uganda with an estimated population of 44,800 persons (2012 population estimate) in an area of 38 km^2^. In November 2013, inclusion criteria were expanded to include adult TB patients living in Osukuru sub-county, an adjacent rural area bordering Tororo, due to lower than expected TB incidence in Tororo. Methods for recruitment, enrollment and study procedures have been previously described.[11] All TB treatment in Tororo municipality is provided by six clinics, and by three clinics in Osukuru sub-county, with clinical and medication administration records kept in Uganda Ministry of Health TB registries at each clinic. Study staff conducted routine surveillance at all clinics offering TB treatment in Tororo and Osukuru, with the goal of recruiting and enrolling adult residents at time of TB treatment start. Study staff coordinated with TB clinic staff to receive a phone call whenever an adult initiated TB treatment, and in addition, conducted weekly (in high-volume clinics) or monthly (in low volume clinics) review of TB registries in each clinic to monitor for missed cases.

Each time a TB patient residing in the study communities was identified, study staff would meet the patient at the clinic to review eligibility for study inclusion. Inclusion criteria were age ≥18 years, reported residence in the study communities for ≥1 month prior to TB diagnosis, and confirmed or suspected TB disease initiating treatment. Eligible adults who consented to participate provided written informed consent and were enrolled.

Study staff then conducted a TB case interview to gather demographic and health information, as well as names and demographic information of all household members (defined as persons living under the same roof as the TB case), and of all frequent non-household contacts with which the participant had spent at least 12 hours in total, either in or out of the home, over the two weeks prior to enrollment (**S1 Questionnaire**). Staff asked participants to recall all locations visited for work and health care (not including the visit to initiate TB treatment when enrollment occurred), as well as for social activities (e.g. places of worship, commerce, and entertainment, etc.), where participants spent ≥12 hours in total, over the one-month prior to enrollment (**S2 Questionnaire**). Study staff collected additional clinical information for each participant including HIV antibody test results and CD4^+^ cell count results, if available, from clinic TB registries. Study staff then collected two sputum specimens from each participant, either as spot specimens, or early morning specimens, based on participant preference. Participants who were unable to spontaneously expectorate sputum underwent induction with hypertonic saline.

Study staff then accompanied participants to their home to obtain global positioning system (GPS) coordinates of the household and conduct a household contact investigation. Staff interviewed consenting adult household members to conduct a brief medical questionnaire, to gather names and demographic information of social contacts that had visited the household in the two weeks prior, and to screen for symptoms of active TB (**S3 Questionnaire**). Child contacts (<18 years) were not interviewed, but study staff provided information regarding TB signs and symptoms, and encouraged adult household members to bring any symptomatic children to the local district hospital for evaluation. Any adult household contact reporting current cough, or any recent hemoptysis, fever, weight loss, or night sweats, were advised to seek evaluation for TB at the local district hospital and provided a transport voucher reimbursable at the hospital. Study staff recorded the results of the hospital-based TB evaluation for each household contact.

Sputum samples from TB patients were kept at 4° C and transported to the Makerere University Mycobacteriology laboratory in Kampala, Uganda for direct sputum examination using fluorescence microscopy and *Mycobacterium tuberculosis* (MTB) culture using Lowenstein-Jensen and BACTEC MGIT. MTB DNA isolated from all culture-positive specimens was sent to the San Francisco General Hospital MTB Research Laboratory and to the Microbial Diseases Laboratory of the California Department of Public Health for genotyping. Genotyping was performed using 24-locus *Mycobacterial* interspersed repetitive unit-variable number tandem repeat (MIRU-VNTR) typing and spoligotyping. Participant strains were analyzed using the MIRU-VNTR*plus* web application (http://www.miru-vntrplus.org).[12] Strains that shared identical MIRU-VNTR and spoligotype patterns were considered as belonging to the same genotypic cluster and assumed to belong to a common, recent TB transmission network. We used the “n-1” method to estimate the proportion of TB cases attributable to recent transmission.[13]

To explore potential epidemiologic links between TB cases within each MTB genotypic cluster, we first created social networks of TB cases, their household contacts, and all named non-household contacts. Next, we created social-location networks that shared ties (i.e. network “edges”) between TB cases and specific locations reported for work, health care or socializing.[11] For example, a TB case who reported socializing at a specific bar could be linked in a social-location network to another TB case who reported socializing at that same bar, even if they did not name one another as social contacts. Social networks and social-location networks were visualized using Gephi software (Gephi.org, version 0.9.1). In addition, all GPS locations (household, work, clinical and social sites) for each TB case within each MTB genotypic cluster were mapped using ArcGIS (version 10.4) in order to identify sites of spatial overlap, defined as locations that were within 100 meters of one another. This latter analysis was performed in an effort to identify shared or neighboring locations (such as neighboring households, or a household adjacent to a bar) that might not be recognized by location name alone.

Lastly, to better understand potential factors that contribute to developing TB as a result of recent TB transmission versus reactivation TB, we compared demographic and clinical characteristics of genotype clustered vs. non-clustered TB cases using chi-squared or t-tests, as appropriate. We also analyzed social-location networks of TB cases stratified by genotype clustering status, and compared network density (i.e. the proportion of potential network connections that are actual connections) by location type between clustered vs. non-clustered cases with UCINET (version 6.627), using bootstrapping to generate estimates of variance for network density.

The Makerere University School of Medicine Research and Ethics Committee, the Ugandan National Council on Science and Technology, and the UCSF Committee on Human Research approved the study.

## Results

Over three years, 173 adults were diagnosed with TB disease and initiated on treatment in the study community. Estimated adult TB incidence was 193 cases per 100,000 person-years for Tororo municipality over three years, and 89 cases per 100,000 person-years for Osukuru parish over 18 months. Within Tororo municipality, the number of reported TB cases declined annually over the study period, from 68 cases in 2012–13, to 35 cases in 2013–14, and 27 cases in 2014–15, with an estimated adult TB incidence rate of 304, 156 and 120 cases/100,000 person-years, respectively.

Of 173 TB cases, 131 (76%) adults were enrolled. Demographic and clinical characteristics of enrolled TB cases are shown in **Table 1**(see also **S1 Dataset**). Of 127 (97%) participants that accepted HIV testing at the time of TB treatment initiation, 66 (52%) adults were HIV-infected: 32% (21/66) of HIV-infected adults reported being on antiretroviral therapy (ART) prior to TB diagnosis and treatment. The proportion of HIV-infected adults on ART prior to TB diagnosis increased from 14% (4/29) in 2012–13, to 33% (6/18) in 2013–14, to 58% (11/19) in 2014–15. Among 23 (35%) HIV-infected adults with CD4^+^ cell count results available from clinic records, mean CD4^+^ cell count was 260 cells/μL (range: 5–701).

**Table 1:** Demographic and clinical characteristics of enrolled TB cases initiating treatment from 2012–2015 in Tororo municipality and Osokuru parish, Uganda.

TB Cases (N = 131)	N	*%*
*Demographic Characteristics*		
Age, median (IQR)	36 (29–44)	
Female	48	*37*
Hospitalized at enrollment	21	*16*
Marital Status		
Single	23	*18*
Married	57	*44*
Widowed/Divorced/Separated	51	*39*
Live alone	29	*22*
Residence in Tororo Municipality (vs. Osukuru)	95	*73*
Residence in study community ≥ 6 months prior to TB diagnosis	121	*92*
*Clinical Characteristics*		
Sputum Microscopy* AFB positive	95	*73*
AFB negative	34	*26*
MTB culture positive (104/131 with sputum culture)	84/104	*81*
New TB Diagnosis	116	*89*
Prior TB Diagnosis	15	*11*
Defaulted during past treatment	8	
Completed prior treatment (recurrent TB)	7	
Site of TB Involvement		
Pulmonary TB	121	*92*
Pulmonary & Extrapulmonary TB	7	*5*
Extrapulmonary TB	3	*2*
Cough duration, median	6 weeks	
HIV-infected (N = 127 accepted HIV testing)	66/127	*52*
In HIV care	53/66	*80*
On antiretroviral therapy at enrollment	21/66	*32*
Mean CD4^+^ cell count, cells/μL (range)**	260 (5–701)	
Smoke tobacco	18	*14*
Any alcohol use	76	*58*
Diabetes, self-reported	4	*3*

*2 participants with missing microscopy results

** CD4^+^ count only available for 23/66 (35%) HIV-infected participants

Enrolled TB cases reported a total of 343 household contacts; 23 (18%) of 131 cases reported living alone. Median household size was three persons (IQR: 2–4 persons) among 108 TB cases who did not live alone. Of 343 household contacts, 118 (34%) were adults (≥18 years), the majority of whom (75/118 [71%]) were women; 105/118 (89%) adult household contacts underwent an interview and TB symptom screening. Of the 105 household contacts interviewed, 85 (81%) reported ever testing for HIV, and 20 (19%) self-reported being HIV positive (**S2 Dataset**). Twenty (19%) contacts screened positive for current cough, fever, night sweats or weight loss, with cough as the most common symptom reported in 14 (13%) contacts. Symptoms were more common among HIV-infected contacts (7/20 [35%]) than HIV-uninfected/status unknown contacts (13/85 [15%]). Among symptomatic contacts, 16/20 (80%) accepted a referral voucher for evaluation at the hospital. Of the four symptomatic contacts that declined referral, one had recently initiated TB treatment outside of the study community. Of the 16 household contacts provided a transport voucher, 11 presented for evaluation, of whom three were diagnosed with active TB and enrolled. Therefore, overall 4/105 (4%) adult household contacts were found to have co-prevalent active TB at the time of household contact investigation, with a yield of 3/105 (3%) new active TB diagnoses, and 3/20 (15%) new diagnoses among symptomatic contacts. One of the 13 adult household contacts who could not be found for enrollment and symptom screening presented to care eight months after attempted household investigation, and was diagnosed with active TB and enrolled as a TB index case.

Sputum was collected for mycobacterial culture in 108/131 (82%) of TB index case participants. Of the 23 participants that were unable to produce sputum for culture despite attempted induction with hypertonic saline, four had a diagnosis of extra-pulmonary TB, and 14 had been diagnosed with smear-negative pulmonary TB based on clinical suspicion in local clinics at the time of diagnosis, prior to study enrollment. Of 108 participants with sputum obtained for culture, 104 had valid results (four participant samples were contaminated), and 84 (81%) grew MTB. In summary, among 104 cases with sputum results: 79 (76%) cases were both acid-fast bacilli (AFB) smear-positive and MTB culture-positive, 5 (4.8%) cases were AFB smear-negative and MTB culture-positive, 12 (11.5%) cases were AFB smear-positive and MTB culture-negative, 8 (7.7%) cases were AFB smear-negative and MTB culture negative. Among 27 TB cases without MTB culture results, 4 (15%) had a record of AFB smear positivity prior to study enrollment by local non-study laboratories, and 23 (85%) were clinically defined TB cases without microbiological evidence of disease.

**Fig 1:**
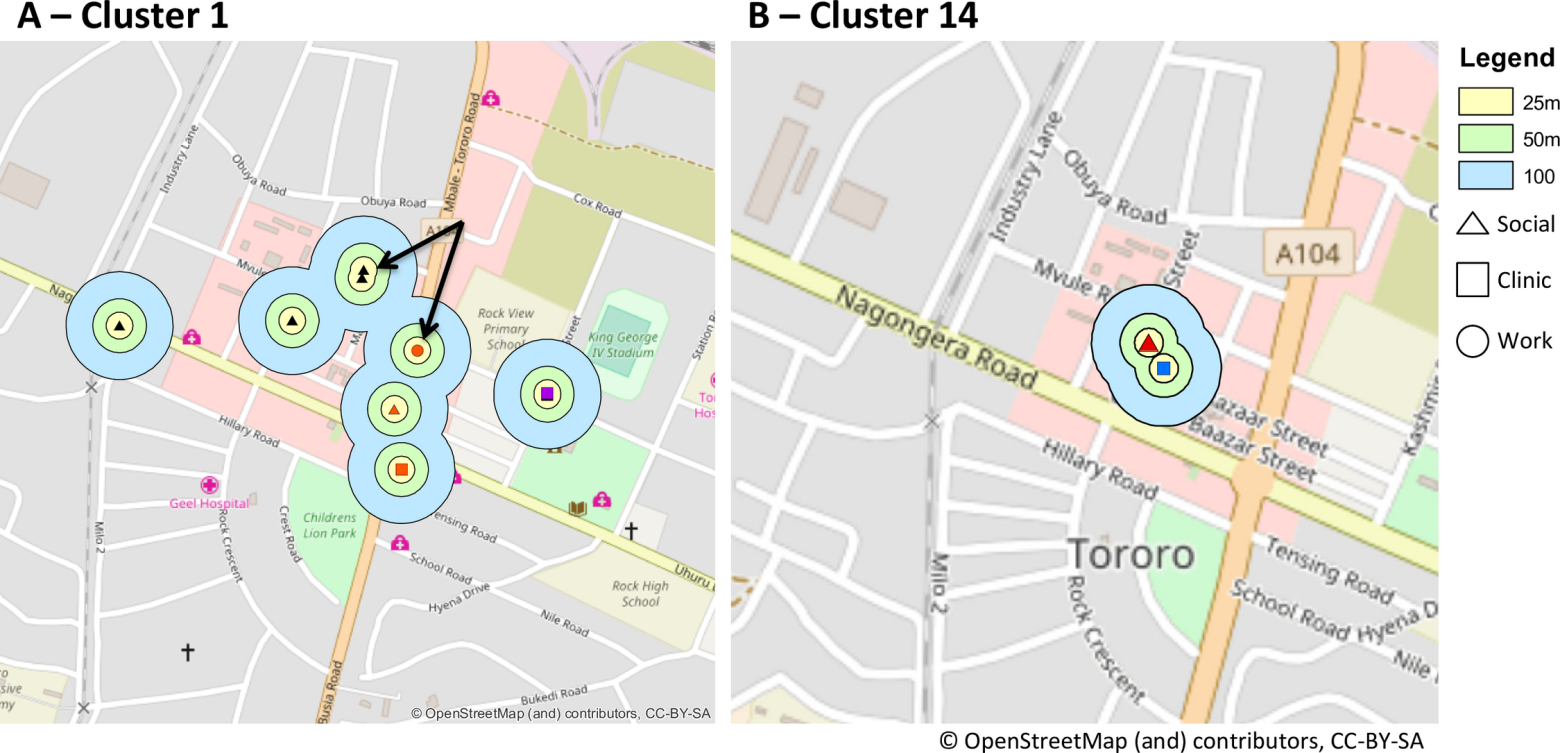
Maps of potential sites of TB transmission within MTB Genotype Clusters 1 and 14, based on geographically adjacent locations of work, healthcare, household or social locations–with each location point color corresponding to a TB patient. Yellow, green and blue circles show buffer zones of 25 meters (m), 50m and 100m respectively, around each mapped location. The arrows in Panel A show that within MTB Genotype Cluster 1, one work location named by a TB patient (red circle) was <100m from two social venues (black triangles) named by another patient. Panel B shows that within Genotype Cluster 14, one TB patient’s clinical location was adjacent (<50m) to a social location visited by another TB patient.

Of the 84 participants with culture-confirmed TB, 33 cases (39%; 95% CI: 29–51%) belonged to 15 distinct MTB genotypic clusters by 24-locus MIRU-VNTR and spoligotyping. Genotypic clusters ranged in size from 2–3 TB cases. The proportion of TB cases attributable to recent transmission by the “n-1” method is 21%. Within each of the 15 genotypic clusters, none of the genotype-matched TB cases shared a household or named one another as non-household social contacts. Of the 4 household contacts who were later enrolled as TB index cases, two were unable to produce sputum for culture, one was MTB culture negative, and one grew MTB on culture with a different genotype than the other household member enrolled. Within each cluster, none of the cases reported any shared non-household contacts. TB cases in six of the 15 clusters reported potential epidemiologic links based on either co-location at a shared site or time spent at geographically adjacent locations (within 100 meters distance), based on GPS coordinates of sites of work, clinic, socializing, and households (**Table 2**and **Fig 1**and **S1 Table**).

**Table 2:** Potential epidemiologic links among patients in six of 15 MTB genotype clusters based on either co-location at a shared site or time spent at geographically adjacent locations (within 100 meters of one another) in the time leading up to TB diagnosis, using GPS coordinates of sites of work, clinic, socializing, and household. Patients in the remaining nine of 15 MTB genotype clusters had no shared or neighboring locations identified.

Cluster Number	TB cases	HIV+ TB Cases	Shared locations	Neighboring locations
1	3	1 of 3	-	Patient A (HIV-) workplace (bank) is <100m from 2 social venues (video hall, church) visited by Patient B (HIV-)
3	2	1 of 2	2 TB cases (one HIV+ and one HIV-) share the same local clinic	-
7	2	2 of 2	-	• Patient A’s workplace (school) is <25m from pharmacy visited by Patient B • Patient A’s household is <50m from pharmacy visited by Patient B • Patient B’s workplace (transport hub) is <25m from social venue (market) visited by Patient A
8	2	None	-	Patient A’s household is <100m from pharmacy and social venue (market) visited by Patient B
14	2	None	-	Patient A’s pharmacy is <50m from social venue (market) visited by Patient B
15	2	1 of 2	2 TB cases share the same local clinic	Patient A’s (HIV-) clinic is <100m from both household and social venue (market) visited by Patient B (HIV+)

**Fig 2:**
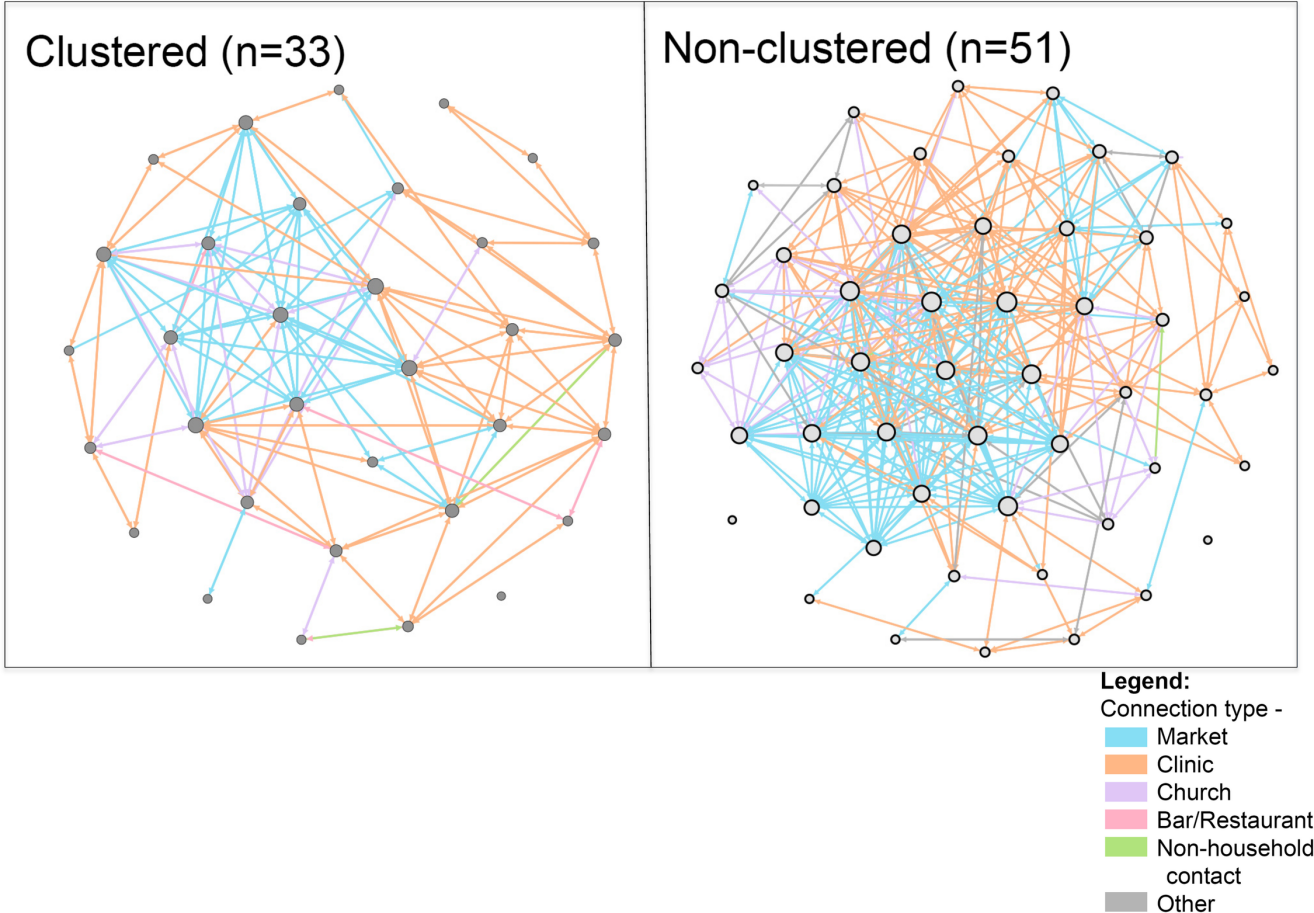
Social-location networks of genotype-clustered vs. non-clustered TB cases in Tororo, Uganda. Each network node represents a participant with culture-positive TB, with node size proportional to node degree. Each network edge represents a specific shared location, with edge colors indicating the location type, as indicated in the legend.

When comparing demographic and clinical characteristics of adults with TB disease within MTB genotype clusters (N = 33) to adults with TB that were not identified as part of a genotypic cluster (i.e. non-clustered TB cases, N = 51), a significantly greater proportion of patients within genotype clusters reported longer cough durations and significantly less tobacco use than non-clustered TB cases in unadjusted analyses (**Table 3**). When examining person-time at location types prior to TB diagnosis, a significantly greater proportion of non-clustered cases (76%) reported spending time socializing at bars/restaurants than clustered cases (55%); otherwise no significant differences in person-time were observed at other location types (**Table 4**). Finally, when examining social networks that linked TB cases to one another based on time spent at specific locations within the community during the study period, stratified by genotype clustering status, genotype-clustered TB case social networks had a significantly greater network density (i.e. greater connectedness of nodes) based on shared medical clinics, and a significantly decreased network density based on shared marketplaces, compared to non-clustered TB case social networks (**Table 5**and **Fig 2**and **S2 Table**).

**Table 3:** Demographic and clinical characteristics of MTB genotype non-clustered vs. clustered TB cases.

TB Cases	Non-clustered N = 51	Clustered N = 33	p-value
*Demographic Characteristics*			
Age, median (IQR)	36 (28–45)	35 (30–34)	0.812
Female	18 (35%)	11 (33%)	1.000
Hospitalized at enrollment	5 (10%)	4 (12%)	0.733
Marital Status			0.630
Single	10 (20%)	4 (12%)	-
Married	26 (51%)	17 (52%)	-
Widowed/Divorced/Separated	15 (29%)	12 (36%)	-
Live alone	13 (25%)	5 (15%)	0.291
Travel via taxi/public bus (“matatu”)	15 (29)	9 (27)	0.832
*Clinical Characteristics*			
Sputum Microscopy			0.377
AFB positive	49 (96%)	30 (91%)	
AFB negative	2 (4%)	3 (9%)	
TB Diagnosis			0.815
New Case	44 (86%)	28 (85%)	
Relapse	3 (6%)	3 (9%)	
Default	3 (6%)	2 (6%)	
Treatment failure	1 (2%)	0 (0%)	
Cough duration			0.012
<2 weeks	0 (0%)	1 (3%)	
2–4 weeks	4 (8%)	1 (3%)	
1–2 months	25 (49%)	6 (18%)	
3–4 months	12 (24%)	15 (46%)	
>4 months	10 (20%)	10 (30%)	
HIV-infected	23 (45%)	12 (36%)	0.714
In HIV care	18 (75%)	10 (77%)	1.000
On antiretroviral therapy at enrollment	7 (29%)	4 (31%)	1.000
Smoke tobacco	12 (57%)	2 (17%)	0.033
Any alcohol use	31 (61%)	16 (48%)	0.368
Diabetes, self-reported	1 (2%)	1 (3%)	1.000

Data are in N (%) unless otherwise specified. P-values derived from chi-square analysis for categorical values or t-test for continuous variables.

**Table 4:** Time spent per location type of non-clustered vs. clustered TB cases.

	Number reporting time spent at location (%)		Median hours/month spent at location (IQR)	
Location type	Non-clustered (N = 51)	Clustered (N = 33)	Non-clustered	Clustered
Clinic^1^	49 (96)	31 (94)	4 (2–10)	4 (2–6)
Church	44 (86)	32 (97)	8 (8–8)	8 (8–8)
Market	40 (78)	29 (88)	40 (16–60)	40 (8–60)
Bar/Restaurant/Vid. Hall	39 (76)	18 (55)*	28 (8–60)	32 (6–40)
Shop	11 (22)	3 (9)	300 (60–420)	300 (152–300)
School	6 (12)	6 (18)	180 (16–300)	150 (120–200)
Transport	4 (8)	1 (3)	420 (300–420)	300 (300–300)
Hotel	4 (8)	4 (12)	28 (10–120)	16 (12–24)
NGO	1 (2)	2 (6)	180 (180–180)	120 (120–120)
Bank	4 (8)	1 (3)	180 (180–180)	64 (6–160)
Farm	1 (2)	1 (1)	420 (420–420)	-
Garage	1 (2)	2 (6)	300 (300–300)	40 (4–300)
Municipal	1 (2)	2 (6)	420 (420–420)	7 (2–12)
Factory	0 (0)	1 (3)	-	300 (300–300)

^1^Including pharmacies.

*p<0.05 compared to non-clustered.

**Table 5:** Comparative network density of MTB genotype-clustered and non-clustered TB cases, within social-location networks (i.e. networks included named locations as nodes). Network density here defined as the proportion of potential connections in a network that are actual connections (i.e. higher density represents a higher connectedness among nodes in a network).

	Non-clustered N = 634*		Clustered N = 241*		p-value**
Connection type	No. (%) connections	Density (SD)	No. (%) connections	Density (SD)	
Clinic	253 (40)	0.108 (0.001)	117 (49)	0.118 (0.006)	<0.001
Market	263 (41)	0.112 (0.015)	82 (34)	0.083 (0.003)	<0.001
Church	68 (11)	0.028 (0.005)	31 (13)	0.030 (0.121)	0.244
Bar/Restaurant/Vid Hall	44 (7)	0.019 (0.015)	9 (4)	0.009 (0.239)	0.110
School	2 (<1)	0.001 (0.000)	0 (0)	-	-
Non-household contact	2 (<1)	0.001 (0.148)	2 (<1)	0.002 (0.141)	0.995

*Total number of connections in each network.

**T-test comparing network densities and bootstrapped standard deviations based on connection type.

## Discussion

In this molecular epidemiologic study of TB transmission in rural Uganda, over one-third of culture-confirmed TB cases initiating treatment belonged to genotype-matched clusters suggestive of recent transmission. With intensive efforts to identify epidemiologic links between members of MTB genotype-clusters, including the use of household contact investigation, social network analysis and GIS analysis of sites of spatial overlap in the community, potential links between TB cases could be identified between cases in 40% (six of 15) of MTB genotypic clusters. These potential epidemiologic links were only identifiable through time spent at shared locations, and health care locations in particular, rather than through named social contacts in this community. This suggests that high rates of transmission events are occurring between “casual” contacts and that nosocomial transmission remains a major source of TB spread in rural Africa, emphasizing the continuing need for improved infection control measures in health care settings.

The finding that 39% of participants with culture-confirmed TB disease belonged to genotype-matched clusters falls within range of prior estimates from east Africa,[14, 15] and lower than estimates of recent transmission from southern Africa.[2, 4] There are relatively few published estimates of the proportion of incident TB cases attributable to recent transmission from population-wide surveillance in African communities, compared to resource-rich settings.[16] Strengths of our estimate of genotypic clustering include rigorous, routine surveillance at all sites of TB treatment initiation in a well-defined geographic region over three years, and use of 24-loci MIRU-VNTR with spoligotyping. However, our estimate of clustering almost certainly represents an underestimate of recent TB transmission in this community, as many transmission events result in latent TB infection of contacts (that cannot be linked to an index case), not all eligible TB cases in the community could be enrolled, and 18% of study participants were unable to produce sputum for MTB culture.

Potential epidemiologic links between cases could not be identified in 60% of genotype-matched TB clusters in this community despite our use of household contact investigation, ascertainment of social contacts (i.e. non-household social contacts of both index cases and their household members), and GPS mapping of homes, and social, work and clinical locations frequented by TB cases. No direct epidemiologic links between genotype-matched cases were identified via named contacts. Other molecular epidemiologic studies from sub-Saharan Africa have reported similar challenges in identifying epidemiologic links within MTB genotype clusters, as well as low rates of transmission attributable to known, close contacts.[4, 17–19] Indeed, we employed multiple methods to explore potential epidemiologic links between genotype-matched TB cases based on the assumption that these links would be difficult to identify. Our results support the observation that the majority of TB transmission events resulting in TB disease take place outside of households, between “casual” contacts who may not know one another well.

Despite the lack of shared households among genotype-matched TB cases, co-prevalent active TB was relatively common among adult household contacts (4%), and the yield of adult household contact investigation was relatively high (3%) for the detection of secondary TB cases, compared to other methods of active TB case finding.[20] Our estimate of co-prevalent TB is similar to an estimate of 2.6% in a study from Kampala, Uganda, though in this latter study, the majority (69%) of co-prevalent TB cases in household contacts had a genotype-match to the TB index case, whereas 22% grew MTB with a different genotype.[21] Our inability to detect shared genotypes among household contacts later diagnosed with active TB likely reflects the low absolute number of contacts (4) with co-prevalent TB; only one of the four contacts diagnosed with active TB grew MTB in culture and this contact’s genotype differed from the TB index case. In addition, the majority of household contacts were children (66%) and not eligible for enrollment in our study, though adults were encouraged to bring symptomatic children to the local hospital for evaluation. In spite of the lack of household epidemiologic links among genotype-clustered cases in our study, household contact investigation offers a high-yield strategy for active TB case finding and an opportunity for TB preventive therapy among latently infected household contacts.

TB cases belonging to genotype clusters had greater network densities based on shared clinics compared to non-clustered cases, suggesting a relatively greater contribution of clinical settings in promoting recent TB transmission within this community compared to other location types. Conversely, the higher rates of tobacco use and the greater person-time spent at drinking venues observed among non-clustered TB cases may reflect either higher rates of reactivation TB among persons engaging in tobacco and alcohol use in this setting,[22, 23] or could result from lower case detection in these populations, resulting in misclassification of persons belonging to recent transmission networks as non-clustered (i.e. reactivation disease) TB cases. We did not observe an association between HIV and genotypic clustering in our study population, though over half of TB cases treated in this rural Ugandan community were HIV-infected.

TB incidence declined steadily over the three years of the study. There were no changes in TB diagnostic capacity at clinics in the study community over this time period. Similar declines in TB case detection rates have been reported across east Africa.[24, 25] These declines may be a consequence of increased antiretroviral therapy (ART) use among HIV-infected persons in Uganda, and indeed ART use among TB cases in our study population increased greater than 4-fold (from 14% to 58%) over the study period. The impact of continued ART expansion on TB incidence and transmission dynamics, with Ugandan guidelines recommending ART for all HIV-infected persons as of early 2017,[26] merits further study.

Our study has several limitations. Incomplete sampling can result in underestimation of the proportion of incident TB cases attributable to recent transmission, in part due to misclassification of TB cases as “non-clustered” that truly occurred within recent TB transmission networks. Several factors that contributed to incomplete sampling in this study included incomplete case detection at local clinics, incomplete enrollment of diagnosed TB cases, an inability to collect sputum from all study participants, and using data from three years of TB surveillance in this community rather than a longer time frame. Continuing enrollment for a longer time period may have reduced “clustered” vs. “non-clustered” TB case misclassification.[27] The incomplete sampling and study attrition observed in our study likely introduced selection bias, and may have resulted in an inaccurately low estimation of incident TB due to recent transmission and in missed opportunities to identify sites of TB transmission. Despite these limitations, our surveillance system at all TB clinics within the study community was designed to reduce incomplete sampling, and though our study sample size of MTB culture-positive cases was relatively small compared to TB molecular epidemiologic studies in resource-rich settings, ultimately greater than three-quarters of TB cases detected in the study community over three years were enrolled. In addition, most participants that could not provide sputum had extra-pulmonary TB or were AFB smear-negative sputum prior to study enrollment, and therefore may have been relatively less likely to contribute to ongoing TB transmission networks than participants that provided sputum samples. In order to facilitate participant recall and based on the assumption that social contacts in a rural setting would be stable over time, we asked participants to provide named social contacts for the two weeks prior to enrolment. This may have resulted in missing epidemiologic links between TB cases within genotype-clusters, and may explain why 60% of genotype-clustered cases had no identifiable epidemiologic links. Additionally, our investigation of shared locations, particularly neighboring locations based on close proximity (Table 2), may be coincidental and we can only consider these sites “potential” sites of transmission. Finally, whole genome sequencing likely would have provided a more discriminating genotyping method than MIRU-VNTR–the methodology used in this study, and may have identified instances where we misclassified TB cases as genotype-clustered that would not be considered clustered by whole genome sequencing.[28, 29] Furthermore, MTB DNA genotyping methods can only confirm transmission events that result in active disease and that occur between culture-positive cases.

In conclusion, identifying potential epidemiologic links identified between MTB culture-positive, genotype-matched TB patients proved challenging, but possible, in a rural African setting, with a combination of molecular epidemiologic tools, and social network and GIS analyses. Our findings suggest most transmission is occurring between casual contacts, and emphasizes the need for improved infection control in health care settings in rural Africa.

## Supporting information

S1 Questionnaire TB index case questionnaire form. (PDF)

S2 Questionnaire TB index case report form used to collect household and non-household contacts, as well locations frequented outside of the home. (PDF)

S3 Questionnaire Household contact questionnaire form. (PDF)

S1 Dataset TB index questionnaire response, TB registry and laboratory results of AFB smear and MTB culture: De-identified data file. (DTA)

S2 Dataset Household contact questionnaire response: De-identified data file. (DTA)

S1 Table Global positioning system (GPS) coordinates of work, clinic and social locations reported by TB index case participants. (XLSX)

S2 Table Social network analysis links (edges), indicating source node and target node, including locations (de-identified). (XLSX)

## References

[1] World Health Organization (WHO). Global Tuberculosis Report 2013. Geneva, Switzerland. 2013.

[2] Glynn JR, Crampin AC, Yates MD, Traore H, Mwaungulu FD, Ngwira BM, et al The importance of recent infection with Mycobacterium tuberculosis in an area with high HIV prevalence: a long-term molecular epidemiological study in Northern Malawi. The Journal of infectious diseases. 2005;192(3):480–7. doi: 10.1086/431517 15995962 10.1086/431517

[3] Verver S, Warren RM, Munch Z, Vynnycky E, van Helden PD, Richardson M, et al Transmission of tuberculosis in a high incidence urban community in South Africa. International journal of epidemiology. 2004;33(2):351–7. doi: 10.1093/ije/dyh021 15082639 10.1093/ije/dyh021

[4] Middelkoop K, Mathema B, Myer L, Shashkina E, Whitelaw A, Kaplan G, et al Transmission of Tuberculosis in a South African Community With a High Prevalence of HIV Infection. The Journal of infectious diseases. 2014. 10.1093/infdis/jiu403 PMC4334823 25053739

[5] Crampin AC, Glynn JR, Fine PE. What has Karonga taught us? Tuberculosis studied over three decades. Int J Tuberc Lung Dis. 2009;13(2):153–64. 19146741 PMC3272402

[6] Middelkoop K, Bekker LG, Mathema B, Shashkina E, Kurepina N, Whitelaw A, et al Molecular epidemiology of Mycobacterium tuberculosis in a South African community with high HIV prevalence. The Journal of infectious diseases. 2009;200(8):1207–11. doi: 10.1086/605930 19764885 10.1086/605930 PMC2932637

[7] Wilkinson D, Pillay M, Crump J, Lombard C, Davies GR, Sturm AW. Molecular epidemiology and transmission dynamics of Mycobacterium tuberculosis in rural Africa. Tropical medicine & international health: TM & IH. 1997;2(8):747–53. 9294544 10.1046/j.1365-3156.1997.d01-386.x

[8] World Health Organization: Recommendations for investigating contacts of persons with infectious tuberculosis in low- and middle-income countries. 2012. 24404639

[9] Middelkoop K, Bekker LG, Morrow C, Lee N, Wood R. Decreasing household contribution to TB transmission with age: a retrospective geographic analysis of young people in a South African township. BMC infectious diseases. 2014;14(1):221. 24758715 10.1186/1471-2334-14-221 PMC4012060

[10] Verver S, Warren RM, Munch Z, Richardson M, van der Spuy GD, Borgdorff MW, et al Proportion of tuberculosis transmission that takes place in households in a high-incidence area. Lancet. 2004;363(9404):212–4. doi: 10.1016/S0140-6736(03)15332-9 14738796 10.1016/S0140-6736(03)15332-9

[11] Chamie G, Wandera B, Marquez C, Kato-Maeda M, Kamya MR, Havlir DV, et al Identifying locations of recent TB transmission in rural Uganda: a multidisciplinary approach. Tropical medicine & international health: TM & IH. 2015. 10.1111/tmi.12459 PMC4355181 25583212

[12] Weniger T, Krawczyk J, Supply P, Niemann S, Harmsen D. MIRU-VNTRplus: a web tool for polyphasic genotyping of Mycobacterium tuberculosis complex bacteria. Nucleic Acids Res. 2010;38(Web Server issue):W326–31. doi: 10.1093/nar/gkq351 20457747 10.1093/nar/gkq351 PMC2896200

[13] Small PM, Hopewell PC, Singh SP, Paz A, Parsonnet J, Ruston DC, et al The epidemiology of tuberculosis in San Francisco. A population-based study using conventional and molecular methods. The New England journal of medicine. 1994;330(24):1703–9. doi: 10.1056/NEJM199406163302402 7910661 10.1056/NEJM199406163302402

[14] Asiimwe BB, Joloba ML, Ghebremichael S, Koivula T, Kateete DP, Katabazi FA, et al DNA restriction fragment length polymorphism analysis of Mycobacterium tuberculosis isolates from HIV-seropositive and HIV-seronegative patients in Kampala, Uganda. BMC infectious diseases. 2009;9:12 doi: 10.1186/1471-2334-9-12 19196450 10.1186/1471-2334-9-12 PMC2645406

[15] Tessema B, Beer J, Merker M, Emmrich F, Sack U, Rodloff AC, et al Molecular epidemiology and transmission dynamics of Mycobacterium tuberculosis in Northwest Ethiopia: new phylogenetic lineages found in Northwest Ethiopia. BMC infectious diseases. 2013;13:131 doi: 10.1186/1471-2334-13-131 23496968 10.1186/1471-2334-13-131 PMC3605317

[16] Mears J, Abubakar I, Cohen T, McHugh TD, Sonnenberg P. Effect of study design and setting on tuberculosis clustering estimates using Mycobacterial Interspersed Repetitive Units-Variable Number Tandem Repeats (MIRU-VNTR): a systematic review. BMJ Open. 2015;5(1):e005636 doi: 10.1136/bmjopen-2014-005636 25609667 10.1136/bmjopen-2014-005636 PMC4305070

[17] Glynn JR, Guerra-Assuncao JA, Houben RM, Sichali L, Mzembe T, Mwaungulu LK, et al Whole Genome Sequencing Shows a Low Proportion of Tuberculosis Disease Is Attributable to Known Close Contacts in Rural Malawi. PLoS ONE. 2015;10(7):e0132840 doi: 10.1371/journal.pone.0132840 26181760 10.1371/journal.pone.0132840 PMC4504505

[18] Surie D, Fane O, Finlay A, Ogopotse M, Tobias JL, Click ES, et al Molecular, Spatial, and Field Epidemiology Suggesting TB Transmission in Community, Not Hospital, Gaborone, Botswana. Emerging infectious diseases. 2017;23(3):487–90. doi: 10.3201/eid2303.161183 27869604 10.3201/eid2303.161183 PMC5382725

[19] Tudo G, Gonzalez-Martin J, Obama R, Rodriguez JM, Franco JR, Espasa M, et al Molecular epidemiology of tuberculosis in the Bata and Malabo districts of Equatorial Guinea. Int J Tuberc Lung Dis. 2004;8(12):1458–63. 15636492

[20] Kranzer K, Houben RM, Glynn JR, Bekker LG, Wood R, Lawn SD. Yield of HIV-associated tuberculosis during intensified case finding in resource-limited settings: a systematic review and meta-analysis. The Lancet infectious diseases. 2010;10(2):93–102. doi: 10.1016/S1473-3099(09)70326-3 20113978 10.1016/S1473-3099(09)70326-3 PMC3136203

[21] Whalen CC, Zalwango S, Chiunda A, Malone L, Eisenach K, Joloba M, et al Secondary attack rate of tuberculosis in urban households in Kampala, Uganda. PLoS ONE. 2011;6(2):e16137 doi: 10.1371/journal.pone.0016137 21339819 10.1371/journal.pone.0016137 PMC3038854

[22] Bates MN, Khalakdina A, Pai M, Chang L, Lessa F, Smith KR. Risk of tuberculosis from exposure to tobacco smoke: a systematic review and meta-analysis. Archives of internal medicine. 2007;167(4):335–42. doi: 10.1001/archinte.167.4.335 17325294 10.1001/archinte.167.4.335

[23] Lonnroth K, Williams BG, Stadlin S, Jaramillo E, Dye C. Alcohol use as a risk factor for tuberculosis—a systematic review. BMC public health. 2008;8:289 doi: 10.1186/1471-2458-8-289 18702821 10.1186/1471-2458-8-289 PMC2533327

[24] Saito S, Mpofu P, Carter EJ, Diero L, Wools-Kaloustian KK, Yiannoutsos CT, et al Implementation and Operational Research: Declining Tuberculosis Incidence Among People Receiving HIV Care and Treatment Services in East Africa, 2007–2012. Journal of acquired immune deficiency syndromes (1999). 2016;71(4):e96–e106. 26910387 10.1097/QAI.0000000000000896 PMC5902407

[25] Yuen CM, Weyenga HO, Kim AA, Malika T, Muttai H, Katana A, et al Comparison of trends in tuberculosis incidence among adults living with HIV and adults without HIV—Kenya, 1998–2012. PLoS ONE. 2014;9(6):e99880 doi: 10.1371/journal.pone.0099880 24937804 10.1371/journal.pone.0099880 PMC4061026

[26] Uganda Ministry of Health: Consolidated Guidelines for Prevention and Treatment of HIV in Uganda. 2016.

[27] Murray M. Sampling bias in the molecular epidemiology of tuberculosis. Emerging infectious diseases. 2002;8(4):363–9. doi: 10.3201/eid0804.000444 11971768 10.3201/eid0804.000444 PMC2730247

[28] Walker TM, Ip CL, Harrell RH, Evans JT, Kapatai G, Dedicoat MJ, et al Whole-genome sequencing to delineate Mycobacterium tuberculosis outbreaks: a retrospective observational study. The Lancet Infectious diseases. 2013;13(2):137–46. doi: 10.1016/S1473-3099(12)70277-3 23158499 10.1016/S1473-3099(12)70277-3 PMC3556524

[29] Roetzer A, Diel R, Kohl TA, Ruckert C, Nubel U, Blom J, et al Whole genome sequencing versus traditional genotyping for investigation of a Mycobacterium tuberculosis outbreak: a longitudinal molecular epidemiological study. PLoS medicine. 2013;10(2):e1001387 doi: 10.1371/journal.pmed.1001387 23424287 10.1371/journal.pmed.1001387 PMC3570532

