# Supplementary material for: Spatial overlap links seemingly unconnected genotype-matched TB cases in rural Uganda
Source: PLoS One. 2018 Feb 13;13(2):e0192666. doi: 10.1371/journal.pone.0192666 (PMC5811029; doi:10.1371/journal.pone.0192666)
Supplement: S1 Questionnaire — (PDF) [file pone.0192666.s001.pdf]

TRAP TB Index Questionnaire v1 (English)

Q1. Please enter your (the interviewer's) intials: __ __ __

Enter the date of the interview _______ / ________ / ____________ (mm / dd/ yyyy)

Welcome to the TRAP TB (K23) Tororo TB Case Finding Study - INDEX CASE - Interview.

Q2. Enter the participant's Study ID: __ __ __

Q3. For the sake of data integrity, please re-enter the Study Participant's ID

__ __ __ __

9997	Don't Know
9998	Refuse to Answer
9999	Not Applicable

If Q2 is not equal to Q3 then READ: "The Subject IDs do not match. Please re-enter." and skip to Q3.

Q4. At which clinic is this interview taking place? (Choose one)

1	Tororo District Hospital (TDH)
2	TASO Tororo
3	CDC PREP Study (TDH Campus)
4	St. Anthony's Hospital
5	Bison Health Centre III
6	Mudokori Health Centre III
9	Not Applicable

Q5. Is this participant currently an in-patient (i.e. a hospitalized patient)?

1	Yes
0	No
7	Don't Know
8	Refuse to Answer
9	Not Applicable

Q6. Please enter the participant's name (Surname, First name).

__ __

The following questions are to be asked directly to the participant:

Q7. How old are you? __ __ __

997	Don't Know
998	Refuse to Answer

Q8. What is your date of birth? __ __ / __ __ / __ __ __ __ mm / dd / yyyy

2097	Don't Know (Year)
2098	Refuse to Answer (Year)
2099	Not Applicable (Year)

Q9. Please enter the participant's gender:

1	Male
2	Female
7	Don't Know
8	Refuse to Answer
9	Not Applicable

Q10. What is the highest level of education you have completed? (Choose one)

00	No school
01	P1 - P6
02	P7
03	S1 - S3
04	S4
05	S5
06	S6
07	Tertiary/Vocational
08	University
09	Post-graduate
77	Don't Know
88	Refuse to Answer

Q11. What is your marital status? (Choose one)

1	Single
2	Married
3	Widowed
4	Divorced/Separated
88	Refuse to Answer

Q12. What is your occupation? (Choose one)

00	Unemployed
01	Farmer
02	Health Care Worker
03	Market Vendor
04	Shopkeeper
05	Bar owner
06	Transport driver (truck, taxi, boda-boda, bus)
07	Teacher
08	Student
09	Other occupation
77	Don't Know
88	Refuse to Answer

If Q12 is not equal to 9, then skip to Q14.

Q13. Please enter employment description for "other" occupation.

____

Q14. Where do you live within Tororo Municipality? (Choose one)

1	Central TMC
2	Amogoro A or B
3	Nyangole/Kasoli
4	Agururu A or B
5	Bison/Maguria
7	Don't Know
8	Refuse to Answer
9	Not Applicable

Q15.For how long have you lived in Tororo Municipality? (Choose one)

0	All of my life.
1	Other

If Q15 is equal to 0, then skip to Q17.

Q16. Select the length of time the participant has been living in Tororo Municipality. (Choose one)

1	1 - 2 Months
2	3 - 5 Months
3	6 - 12 Months
4	1 - 2 Years
5	3 - 5 Years
6	>5 Years
77	Don't Know
88	Refuse to Answer

Q17. Where were you born? (Choose one)

01	Tororo Municipality (TMC)
02	Tororo District, not TMC
03	Kampala
04	Other Ugandan District (not Kampala or Tororo)
05	Kenya
06	Rwanda
07	Tanzania
08	Ethiopia
09	Sudan (North or South)
10	Congo
11	Somalia
12	Other
77	Don't Know
88	Refuse to Answer

If Q17 is not equal to 12, then skip to Q19.

Q18. Please enter the other place of birth:

____

Q19. What is your ethnicity? (Choose one)

01	Ateso
02	Jopadhola
03	Ganda
04	Soga
05	Samia
06	Gishu
07	Other
97	Don't Know
98	Refuse to Answer

If Q19 is not equal to 7, then skip to Q21.

Q20. Enter participants ethnicity: ____________________

Q21. Have you ever lived outside of Tororo Municipality for more than 1 year?

1	Yes
0	No
7	Don't Know
8	Refuse to Answer

Q22. Have you ever stayed outside of Tororo Municipality for >1 month in the past year?

1	Yes
0	No
7	Don't Know
8	Refuse to Answer

If Q22 is not equal to 1, then skip to instruction before Q24.

Q23. For how long were you outside of Tororo Municipality in the past year? (Choose one)

1	1 - 2 Months
2	3 - 5 Months
3	6 - 11 Months
4	>11 Months
77	Don't Know
88	Refuse to Answer

The next section of questions will address the presence or absence of symptoms for the participant.

Q24. Have you had any fever in the past week?

1	Yes
0	No
7	Don't Know
8	Refuse to Answer

If Q24 is not equal to 1, then skip to Q26.

Q25. For how long have you had a fever? (Choose one)

1	<1 Week
2	1 - 2 Weeks
3	3 - 4 Weeks
4	1 - 2 Months
5	3 - 4 Months
6	> 4 Months
7	Don't Know
8	Refuse to Answer

Q26. Do you have a cough?

1	Yes
0	No
7	Don't Know
8	Refuse to Answer

If Q26 is not equal to 1, then skip to Q29.

Q27. For how long have you had a cough? (Choose one)

0	< 2 Weeks
1	2 - 4 Weeks
2	1 - 2 Months
3	3 - 4 Months
4	> 4 Months
77	Don't Know
88	Refuse to Answer

Q28. Have you had any blood in the mucous (sputum) you cough up?

1	Yes
0	No
7	Don't Know
8	Refuse to Answer

Q29. Are you having sweats at night?

1	Yes
0	No
7	Don't Know
8	Refuse to Answer

If Q29 is not equal to 1, then skip to Q31.

Q30. For how long have you been having sweats at night? (Choose one)

1	< 1 Week
2	1 - 2 Weeks
3	3 - 4 Weeks
4	1 - 2 Months
5	3 - 4 Months
6	> 4 Months
77	Don't Know
88	Refuse to Answer

Q31. Have you lost any weight in the past 3 months?

1	Yes
0	No
7	Don't Know
8	Refuse to Answer

Q32. For how long have you been feeling sick? (Choose one)

0	I don't feel ill.
1	< 1 Week
2	1 - 2 Weeks
3	3 - 4 Weeks
4	1 - 2 Months
5	3 - 4 Months
6	>4 Months
77	Don't Know
88	Refuse to Answer

The next set of questions will ask about the participant's medical history.

Q33. Have you ever been diagnosed with TB (prior to your current TB diagnosis)?

1	Yes
0	No
7	Don't Know
8	Refuse to Answer

If Q33 is not equal to 1, then skip to Q38.

Q34. When were you diagnosed with TB in the past?

__ __ __ __	yyyy
2097	Don't Know (Year)
2098	Refuse to Answer (Year)
2099	Not Applicable (Year)

Q35. Have you ever received treatment (pills) for TB in the past?

1	Yes
0	No
7	Don't Know
8	Refuse to Answer

If Q35 is not equal to 1, then skip to Q38.

Q36. Have you ever started TB pills (antibiotic treatment) in the past and completed the full 8 month course of treatment?

1	Yes
0	No
7	Don't Know
8	Refuse to Answer

Q37. Have you ever started TB pills (antibiotic treatment) in the past but failed to complete the full 8 month course (defaulted)?

1	Yes
0	No
7	Don't Know
8	Refuse to Answer

Q38. Have you ever smoked tobacco regularly?

1	Yes
0	No
7	Don't Know
8	Refuse to Answer

If Q38 is not equal to 1, then skip to Q40.

Q39. Do you currently smoke tobacco?

1	Yes
0	No
7	Don't Know
8	Refuse to Answer

Q40. How often do you have a drink containing alcohol? (Choose one)

0	Never
1	Monthly or less
2	2 - 4 Times per month
3	2 - 3 Times per week
4	4 or more times per week
77	Don't Know
88	Refuse to Answer

If Q40 is equal to 0, then skip to Q43.

Q41. How many standard drinks containing alcohol do you have on a typical day, when drinking? (Choose one)

0	1 or 2 Drinks
1	3 or 4 Drinks
2	5 or 6 Drinks
3	7 - 9 Drinks
4	10 or more Drinks
77	Don't Know
88	Refuse to Answer

Q42. How often do you have six or more drinks on one occasion? (Choose one)

0	Never
1	Less than monthly
2	Monthly
3	Weekly
4	Daily, or almost daily
77	Don't Know
88	Refuse to Answer

Q43. Have you ever been told that you have diabetes?

1	Yes
0	No
7	Don't Know
8	Refuse to Answer

Q44. Have you ever been tested for HIV?

1	Yes
0	No
7	Don't Know
8	Refuse to Answer

If Q44 is equal to 0, then skip to Q46.

Q45. What were the results of your most recent HIV test? (Choose one)

0	HIV Negative
1	HIV Positive
77	Don't Know
88	Refuse to Answer

If Q45 is equal to 0, then skip to instruction before Q52.

Q46. Are you currently in care at an HIV clinic?

1	Yes
0	No
7	Don't Know
8	Refuse to Answer

If Q46 is equal to 0, then skip to instruction before Q48.

Q47. At which clinic do you receive HIV care? (Choose one)

1	Tororo District Hospital (TDH) HIV Clinic
2	TASO Tororo
3	Other
7	Don't Know
8	Refuse to Answer

If Q47 is not equal to 3, then skip to Q49.

Q48. Enter other clinic where participant receives HIV care:

————————————————————————

Q49. Are you taking any HIV medications, other than cotrimoxazole (Septrin)?

1	Yes
0	No
7	Don't Know
8	Refuse to Answer

If Q49 is equal to 0, then skip to Q51.

Q50. Which of the following HIV medications are you taking? (Check all that apply)

__	Tri-immune
__	Combivir (AZT/3TC)
__	Truvada (TDF/FTC)
__	Stavudine (D4T)
__	Lamivudine (3TC)
__	Nevirapine (NVP)
__	Efavirenz (EFV)
__	Kaletra (Lopinavir/ritonavir)
__	Don't Know
__	Refuse to Answer
__	Not Applicable

Q51. Are you taking a medication called Septrin (also known as cotrimoxazole)?

1	Yes
0	No
7	Don't Know
8	Refuse to Answer

We are now going to ask you about your home and to list the members of your household. We are interested in knowing about everyone who has lived with you in your home for at least one month in the past year.

Enter Household Names and Locations on the TRAP TB Enumeration Form.

Q52. How many rooms does your household have? __ __

97	Don't Know
98	Refuse to Answer
99	Not Applicable

Q53. How many of these rooms are used for sleeping? __ __

97	Don't Know
98	Refuse to Answer
99	Not Applicable

Q54. Do you live alone in your home?

1	Yes
0	No
7	Don't Know
8	Refuse to Answer
9	Not Applicable

Q314. Do you do any work outside of your home?

1	Yes
0	No
7	Don't Know
8	Refuse to Answer
9	Not Applicable
8	Refuse to Answer

Q405. How much time do you spend in taxis (Matatus) each week? (Choose one)

01	Daily taxi rides from Monday-Friday
02	3-4 times each week
03	1-2 times each week
04	2-3 times per month
05	Once per month
06	Less than once per month
07	Never
97	Don't Know
98	Refuse to Answer

Q406. How do you get around Tororo Municipality? (Check all that apply)

__	By foot (walking)
__	Bicycle taxi
__	Boda-boda (motorcycle taxi)
__	Matatu (taxi)
__	Private car/motorcycle/bicycle
__	Don't Know
__	Refuse to Answer

The following questionnaire information should now be collected from the TB Clinic Registry. Afterwards, the participant will provide two sputum samples and be brought back to their home.

Q407. (TB Registry) What is the participant's TB treatment status? (Choose one)

1	New Case
2	Relapse
3	Default
4	Treatment Failure
7	Not recorded/Unknown (confirm with TB nurse)

Q408. (TB Registry) TB Registry sputum AFB Smear result (Choose one)

0	Smear Negative
1	Smear Positive
7	Not recorded/Unknown (Confirm with nurse)

Q409. (TB Registry) Does this patient have a diagnosis of Extrapulmonary TB?

1	Yes
0	No

If Q409 is equal to 0, then skip to Q412.

Q410. (TB Registry) What is the anatomic site of extrapulmonary TB? (Choose one)

01	Lymph Node (adenitis)
02	Pleura
03	Meningitis (CNS TB)
04	Spinal
05	Abdominal
06	Uro-genital
07	Other
97	Don't Know
98	Refuse to Answer
99	Not Applicable

If Q410 is not equal to 7, then skip to Q412.

Q411. Enter other site of extrapulmonary TB:

____

Q412. (TB Registry) Please enter the Chest X-ray results: (Choose one)

0	Normal
1	Abnormal (no cavity)
2	Abnormal with cavity
3	No chest x-ray done (confirm with TB nurse)

Q413. (TB Registry) What TB antibiotics are being started at this clinic visit? (Choose one)

1	Isoniazid(H)/ Rifampicin(R)/ Ethambutol(E)/ Pyrazinamide(Z, or PZA)
2	Isoniazid(H)/ Rifampicin(R)/ Ethambutol(E)/ Pyrazinamide (Z, or PZA) and Streptomycin
3	Other regimen
7	Don't Know

If Q413 is not equal to 3, then skip to Q415.

Q414. (TB Registry) List other antibiotic regimen started at this TB clinic visit:

____

Q415. (TB Registry) HIV Test Result: (Choose one)

0	Negative
1	Positive
2	Indeterminate test result
3	Unknown, and HIV testing not available at TB clinic
8	Refuse to Answer

The TRAP TB Tororo Interview is now complete. Proceed to sputum collection.

Q416. (Question for TRAP TB Research Assistant): Was sputum collection successful? (Choose one)

0	No - sputum could not be collected or induced
1	Yes - sputum was collected without induction
2	Yes - sputum was collected with induction
3	Unable to collect - Participant refused sputum collection or induction

Please thank the participant for his/her time, and then provide him/her a ride to their home (or if they decline a ride home, determine where they live in Tororo Municipality and when you can visit their home).
