# Supplementary material for: Spatial overlap links seemingly unconnected genotype-matched TB cases in rural Uganda
Source: PLoS One. 2018 Feb 13;13(2):e0192666. doi: 10.1371/journal.pone.0192666 (PMC5811029; doi:10.1371/journal.pone.0192666)
Supplement: S2 Questionnaire — (PDF) [file pone.0192666.s002.pdf]

The "Tororo TB Case Finding" Study

TB Questionnaire #2 – Index Patient

Interviewer's Initials ______

TB Index Patient

	**Question**	**Response (fill in legibly in ink)**
1	Today's date:	______ / ______ / _______<br>(day) / (month) / (year)
2	Study ID:	__ __ __ - 00

Section 1a. Current Household Members of Index Patient

Household members are defined as people who live together (under the same physical roof) *for at least 1 of the past 12 months*. The only people you should include who have been there for less than 1 month are infants and newly added spouses. The head of the household is always a member and should be listed on the FIRST line, even if he or she has been there for less than 1 month. If the head of the household is the Index case, write "INDEX" under the name and cross-out the ID code.

1. ID Code	2. NAME (surname, given name)<br><br>If the head of the household is the Index case, write "INDEX" under the name and cross-out the ID code.	3. Sex of NAME<br><br>[1] Male<br>[2] Female	4. Relationship of [NAME] to Index Case<br><br>** See codes next page*	5. Current Age		6. Does [NAME] sleep in the same room as Index Case?<br><br>[0] No<br>[1] Yes
				a. Years	b. Mths<br><br>**if age <2 yrs.**	
___ ___ ___ -01						
___ ___ ___ -02						
___ ___ ___ -03						
___ ___ ___ -04						
___ ___ ___ -05						
___ ___ ___ -06						
___ ___ ___ -07						
___ ___ ___ -08						
___ ___ ___ -09						
___ ___ ___ -10						
___ ___ ___ -11						
___ ___ ___ -12						
___ ___ ___ -13						
___ ___ ___ -14						
___ ___ ___ -15						
___ ___ ___ -16						
___ ___ ___ -17						
___ ___ ___ -18						
___ ___ ___ -19						
___ ___ ___ -20						

Relationship of Household Members to Index Case

2 Spouse
3 Son/Daughter
5 Father/Mother
7 Grandson/Granddaughter
9 Grandfather/Grandmother
11 Brother/Sister
13 Uncle/Aunt
15 Co-wife
16 Cousin
17 Niece/Nephew
19 Stepchild
20 Foster child
21 Girlfriend/Boyfriend
------
30 Local Friend/Neighbor
31 Non-local Friend
32 LC chairperson
33 Househelper
34 Employee
35 Employer
36 Landlord
37 Tenant/Renter
38 Priest
40 Healthcare worker
------
44 Other

Section 2. Frequent (Non-Household) Contacts of Index Case

Frequent Contacts (apart from household members) are defined as people, aside from household members, *with whom the Index Case has spent >12 hours in total over the past one month*.

1. ID Code	2. NAME (surname, given name)	3. Sex of NAME<br>[1] Male<br>[2] Female	4. Relationship of [NAME] to Index Case<br>* *See codes below*	Frequency of contact:<br>[1] Daily<br>[2] >3 days/week<br>[3] Weekly<br>[4] Occasional (Less than weekly)	Has this contact been ill (losing weight, coughing) or recently been diagnosed or treated for TB?<br>[0] No<br>[1] Yes<br>[9] Don't know
___ ___ ___ -21					
___ ___ ___ -22					
___ ___ ___ -23					
___ ___ ___ -24					
___ ___ ___ -25					
___ ___ ___ -26					
___ ___ ___ -27					
___ ___ ___ -28					
___ ___ ___ -29					
___ ___ ___ -30					
___ ___ ___ -31					
___ ___ ___ -32					
___ ___ ___ -33					
___ ___ ___ -34					
___ ___ ___ -35					
___ ___ ___ -36					
___ ___ ___ -37					
___ ___ ___ -38					
___ ___ ___ -39					
___ ___ ___ -40					

RELATIONSHIP *TO INDEX PATIENT* CODES

1 Spouse	4 Other relative	7 Employer	10 Health Care Worker	13 Teacher	16 Trader	19 Landlord
2 Mother/Father	5 Friend	8 Employee	11 Traditional Healer	14 Priest	17 Client	44 Other
3 Daughter/Son	6 Neighbor	9 Co-worker	12 Local Councilor (LC)	15 Girlfriend/Boyfriend	18 Househelper	

Section 3a. Locations Visited by Index Case

Work Location: Defined as any place where the Index Case worked in the past three months.
Clinic Location: Defined as any place where the Index Case has gone for medical care in the past three months.
Social Location: Defined as any place where the Index Case spent **12 hours or more** in total over the **past month**.

Location ID	2. Name of LOCATION<br><br>(examples: "Tororo Cement Factory," or "Prime Hotel" or "TDH Male Ward")	3. How often did you go to [LOCATION] in the past month?<br>[1] Daily<br>[2] 3-5 times/week<br>[3] 1-2 times/week<br>[4] Weekly<br>[5] 2-3 times/month<br>[6] Once<br>[7] I did not go to this place in the past month.	4. When you visited [LOCATION], how much time did you spend there?<br><br>[1] >12 hours<br>[2] 8-12 hours<br>[3] 4-8 hours<br>[4] <4 hours	5. Type of social location<br>[1] Restaurant<br>[2] Bar/Club<br>[3] Market place<br>[4] Shop<br>[5] Church/Mosque<br>[6] Hotel/Inn<br>[7] Private Residence<br>[8] Other<br>[9] Ajona or Malwa (local brew drinking circle)<br>[10] Money circle
Category: Household location __ __ __ .50				
Category: Work Locations				
__ __ __ .61				
__ __ __ .62				
__ __ __ .63				
__ __ __ .64				
__ __ __ .65				
Category: Clinic Locations – "Where do you go for medical care?"				
__ __ __ .71				
__ __ __ .72				
__ __ __ .73				
Category: Social Locations				
__ __ __ .81				
__ __ __ .82				
__ __ __ .83				
__ __ __ .84				
__ __ __ .85				
__ __ __ .86				
__ __ __ .87				
__ __ __ .88				
__ __ __ .89				
__ __ __ .90				
