# Supplementary material for: Spatial overlap links seemingly unconnected genotype-matched TB cases in rural Uganda
Source: PLoS One. 2018 Feb 13;13(2):e0192666. doi: 10.1371/journal.pone.0192666 (PMC5811029; doi:10.1371/journal.pone.0192666)
Supplement: S3 Questionnaire — (PDF) [file pone.0192666.s003.pdf]

TRAP TB Contact Screen v1 (English)

Q1. Please enter your (the interviewer's) intials: __ __ __

Q2. Please enter the INDEX CASE Study ID: __ __ __

Q3. To ensure data integrity, please re-enter the Index Case's ID.

__ __ __ __

9997	Don't Know
9998	Refuse to Answer
9999	Not Applicable

Q4. Please enter the HOUSEHOLD CONTACT's Study ID (1-20: based on the household contact enumeration form: for example, the first contact enumerated has an ID of 1; and the tenth contact enumerated has an ID of 10)

__ __

97	Don't Know

Q5. For the sake of data integrity, please re-enter the Household Contact's ID number.

__ __

97	Don't Know

Q6. Please enter the name of the Household Contact [Response to Q4]:

__ __ __ __ __ __ __ __ __ __ __ __ __ __ __ __ __ __ __ __

Section 1. TB Symptom Screening.

Q7.Do you currently have a **cough**?

1	Yes
0	No
7	Don't Know
8	Refuse to Answer

If Q7 is equal to 0, then skip to Q10.

Q8. For how much time have you had a cough? (Choose one)

1	<2 weeks
2	2-4 weeks
3	1-3 months
4	3-6 months
5	>6 months
7	Don't Know
8	Refuse to Answer

Q9. Is their blood in your sputum (mucous) when you cough?

1	Yes
0	No
7	Don't Know
8	Refuse to Answer
9	Not Applicable

Q10. Have you had a **fever** in the past week?

1	Yes
0	No
7	Don't Know
8	Refuse to Answer

Q11. Have you **lost weight** over the past month?

1	Yes
0	No
7	Don't Know
8	Refuse to Answer

Q12. Are you having any sweats at night?

1	Yes
0	No

7 Don't Know
8 Refuse to Answer

Section 2. Household Contact Medical History

Q13. Have you ever been diagnosed with tuberculosis (TB)?
1 Yes
0 No
7 Don't Know
8 Refuse to Answer

If Q13 is not equal to 1, then skip to Q15.

Q14. When were you diagnosed with tuberculosis (TB)?

__ __ / __ __ __ __ mm / yyyy
2097 Don't Know (Year)
2098 Refuse to Answer (Year)
2099 Not Applicable (Year)

Q15.Have you ever been treated for tuberculosis (TB)?
1 Yes
0 No
7 Don't Know
8 Refuse to Answer

Q16. Have you ever smoked tobacco regularly?
1 Yes
0 No
7 Don't Know
8 Refuse to Answer

If Q16 is not equal to 1, then skip to Q18.

Q17. Do you currently smoke tobacco?
1 Yes
0 No
7 Don't Know
8 Refuse to Answer

Q18. Have you ever been tested for HIV?
1 Yes
0 No
7 Don't Know
8 Refuse to Answer

If Q18 is equal to 0, then skip to end of questionnaire.

Q19. What were the results of your most recent HIV test? (Choose one)
0 HIV Negative
1 HIV Positive
77 Don't Know
88 Refuse to Answer

If Q19 is equal to 0, then skip to end of questionnaire.

Q20. Are you currently in care at an HIV clinic?
1 Yes
0 No
7 Don't Know
8 Refuse to Answer

If Q20 is equal to 0, then skip to instruction before Q22.

Q21. At which clinic do you receive HIV care? (Choose one)
1 Tororo District Hospital (TDH) HIV Clinic
2 TASO Tororo
3 Other

7	Don't Know
8	Refuse to Answer

If Q21 is not equal to 3, then skip to Q23.

Q22. Enter other clinic where participant receives HIV care:

____

Q23. Are you taking any HIV medications, other than cotrimoxazole (Septrin)?

1	Yes
0	No
7	Don't Know
8	Refuse to Answer

If Q23 is equal to 0, then skip to end of questionnaire.

Q24. Which of the following HIV medications are you taking? (Check all that apply)

__	Tri-immune
__	Combivir (AZT/3TC)
__	Truvada (TDF/FTC)
__	Stavudine (D4T)
__	Lamivudine (3TC)
__	Nevirapine (NVP)
__	Efavirenz (EFV)
__	Kaletra (Lopinavir/ritonavir)
__	Don't Know
__	Refuse to Answer
__	Not Applicable

If Q7 is equal to 1 or Q10 is equal to 1 or Q11 is equal to 1 or Q12 is equal to 1, then based on the information provided by the household contact, [Response to Q6] should be referred to a medical clinic for further TB evaluation (including sputum microscopy).

The Household Contact interview for [Response to Q6] is now complete. Reopen a new Household Contact questionnaire to perform TB symptom screening on the next household contact of Index Case [Response to Q2]. Thank you.
